# Inhibition of TANK‐binding kinase1 attenuates the astrocyte‐mediated neuroinflammatory response through YAP signaling after spinal cord injury

**DOI:** 10.1111/cns.14170

**Published:** 2023-04-10

**Authors:** Wenbin Zhang, Mengxian Jia, Jiashu Lian, Sheng Lu, Jian Zhou, Ziwei Fan, Zhoule Zhu, Yaozhi He, Changgang Huang, Mingyu Zhu, Jian Wang, Ying Wang, Zhihui Huang, Honglin Teng

**Affiliations:** ^1^ Department of Orthopedics (Spine Surgery) The First Affiliated Hospital of Wenzhou Medical University Wenzhou Zhejiang China; ^2^ School of Pharmacy, and Department of Neurosurgery The Affiliated Hospital, Hangzhou Normal University Hangzhou Zhejiang China; ^3^ Center for Wound Repair and Regeneration The First Affiliated Hospital of Wenzhou Medical University Wenzhou Zhejiang China; ^4^ Department of Clinical Research Center Affiliated Hangzhou First People's Hospital, Zhejiang University School of Medicine Hangzhou Zhejiang China

**Keywords:** amlexanox, astrocytes, noncanonical NF‐κB signaling pathway, spinal cord injury, TBK1, YAP

## Abstract

**Aims:**

TANK‐binding kinase 1 (TBK1) is involved in regulating the pathological process of a variety of inflammatory diseases in the central nervous system. However, its role and underlying molecular mechanisms in spinal cord injury (SCI) remain largely unknown.

**Methods:**

We employed the TBK1 inhibitor amlexanox (ALX) to address this question. An in vivo clip‐compressive SCI model and in vitro lipopolysaccharide (LPS)‐induced astrocyte inflammation model were established to examine the effects of TBK1 inhibition on the expression of proinflammatory cytokines.

**Results:**

In this study, we found that TBK1 and TBK1‐medicated innate immune pathways, such as TBK1/IRF3 and noncanonical NF‐κB signaling, were activated in astrocytes and neurons after SCI. Furthermore, inhibition of TBK1 by ALX alleviated neuroinflammation response, reduced the loss of motor neurons, and improved the functional recovery after SCI. Mechanistically, inhibition of TBK1 activity promoted the activation of the noncanonical NF‐κB signaling pathway and inhibited p‐IRF3 activity in LPS‐induced astrocytes, and the TBK1 activity was required for astrocytic activation through yes‐associated protein (YAP) signaling after SCI and in LPS‐induced astrocytes inflammation model.

**Conclusion:**

TBK1‐medicated innate immune pathway in astrocytes through YAP signaling plays an important role in the pathogenesis of SCI and inhibition of TBK1 may be a potential therapeutic drug for SCI.

## Graphical abstract

ALX treatment inhibits TBK1‐YAP pathway and activates the noncanonical NF‐κB signaling pathway in astrocytes, which results in the suppression of reactive astrocytes, including reduce of cell proliferation, decrease of mRNA level of IL‐6, IL‐1β, reduce of inflammatory cell infiltration, and promotion of the degradation of p100 and the translation of NF‐κB p52‐RELB heterodimers into the nucleus, and inhibition of phosphorylation of IRF3 and reduce of YAP nuclear transfer.

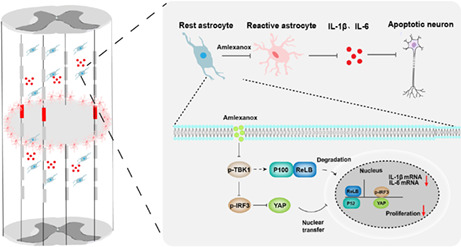

## 1 INTRODUCTION

Spinal cord injury (SCI) is a clinically serious traumatic disease of the central nervous system, and it is one of the main causes of disability worldwide. With the aging of the population and the growth of the population, it affects all health conditions. The total burden caused is increasing year by year.^1^ In daily life, the most common incidents that cause traumatic SCI are traffic accidents (40%–50%), fights (10%–25%), and high falls (20%).^2^ In traumatic SCI, primary injury damages cells and triggers a complex secondary injury cascade, which periodically leads to the death of neurons and glial cells, local vascular injury, and tissue ischemia and edema. Primary injury is irreversible, while secondary injury is a dynamic adjustment process with the infiltration of inflammatory cells.^3^ Therefore, the inflammatory response plays an important role in promoting the pathological process of secondary injury.^4^

The canonical nuclear factor (NF)‐κB signaling pathway has been widely studied in the context of neuroinflammatory responses.^5^, ^6^ Astrocytes generate proinflammatory factors, via the NF‐κB signaling pathway, which is one of the critical contributing factors to secondary injury.^7^ However, the relationship and physiological function of the noncanonical NF‐κB signaling pathway in CNS injury are yet to be defined. The activation of the noncanonical NF‐κB signaling pathway is related to the tightly regulated processing of p100.^8^ The p100/RelB complex exists in an inactive state sequestered in the cytoplasm during nonimmunogen conditions. The activation of p100/RelB complex is initiated by IκB kinase α (IKKα), which phosphorylates carboxy‐terminal serine residues of p100, triggering the generation of p52 and leading the intranuclear translocation of p52 and RelB.^9^ Additionally, TANK‐binding kinase1 (TBK1) induces the production of type I interferon and other cytokines through phosphorylation of interferon regulatory factor 3 (IRF3), which participates in the regulation of innate immunity.^10^ Recent studies have shown that SCI triggers an innate immune response that is a key component of secondary injury pathomechanisms.^11^ Traditionally, the noncanonical NF‐κB pathway has normally been associated with innate immune responses. Therefore, these studies suggest an involvement of TBK1‐medicated innate immune responses in the regulation of CNS inflammation following sterile injury.

Amlexanox (ALX) is commonly used to treat aphthous ulcers, asthma, and allergic rhinitis.^12^ Recent studies have shown that ALX is a specific inhibitor of TBK1,^13^ which regulates the canonical and noncanonical NF‐κB signaling pathway.^14^, ^15^ TBK1 inhibitors exert their neuroprotective effects by reducing the release of high‐mobility group box 1 (HMGB1), which improves neurological functions in ischemic brain damage.^16^ However, it remains unknown that the roles of ALX in SCI, as well as if ALX treatment, could inhibit the activation of astrocyte and the subsequent inflammatory response after SCI.

In the present studies, we found the activation of TBK1‐ and TBK1‐medicated innate immune pathways, such as noncanonical NF‐κB signaling and TBK1/IRF3 pathway from the temporospatial point of view, and ALX attenuated the astrocyte‐mediated neuroinflammatory response involving the interactive roles of IRF3, YAP and the noncanonical NF‐κB following a traumatic SCI.

## 2 MATERIALS AND METHODS

### 2.1 Animals

Adult (25–30 g) male C57BL/6 mice were supplied by Experimental Animal Center of the First Affiliated Hospital of Wenzhou Medical University. As described before,^17^ the *yap* ^*GFAP*^‐CKO conditional knockout mice were generated by crossing the floxed *yap* allele (yap^f/f^) mice with GFAP‐Cre transgenic mice. All mice were kept in a clean, temperature‐controlled with regular light and dark alternation environment with ad libitum access to food and water. All experimental operations were strictly following the Guidelines for the Care and Use of Laboratory Animal from the National Institutes of Health were approved by the ethics commitment of the First Affiliated Hospital of Wenzhou Medical University (Approval 2021‐0139).

### 2.2 SCI model and treatment

The clip‐compressive SCI model was established using a vascular clip as described previously.^17^ In brief, 2‐month male mice were anesthetized with 1.25% avertin (2,2,2‐tribromoethanol, Sigma Millipore) intraperitoneally (20 mL/kg). T8‐T9 laminectomy was performed by mouse spinal cord adapter (RWD, 68094). After exposure of the spinal cord, local anesthesia was applied on the surface of the spinal cord and a vascular clip (30 g force, Oscar) was then used to oppress exposed region for 15 s. The wounds were sutured layer by layer, and animals were placed on a heating pad. After injury, the bladder was manually evacuated thrice daily until the recovery of urinating function. ALX (HY‐B0713, MCE) as a TBK1 inhibitor was first dissolved in 100% DMSO, then dissolved in corn oil to prepare a working solution. Each group of mice was orally administered with ALX (25 mg/kg)^18^ or same volume of vehicle once per day in the first 7 day of SCI.

### 2.3 Locomotion recovery assessment

The Basso Mouse Scale (BMS) locomotor rating scale was utilized to assess the locomotion recovery of SCI mice.^19^ For BMS scoring, hindlimb locomotor performance was evaluated according to the BMS, with scores ranging from 0 (complete paralysis) to 9 (normal mobility), each score represents a distinct motor functional state.

Footprint analysis was used to assess the motor functions of forelimbs and hindlimbs after SCI.^20^ Before testing, all mice were allowed to familiarize the environment for at least 1 h. Then, after each limb was brushed with red (forelimbs) and blue (hindlimbs) nontoxic ink, the animals were put to run along a paper‐lined runway in a dark box. Finally, the step length and width of the mice were qualitatively analyzed.

### 2.4 Lipopolysaccharide (LPS)‐induced astrocyte inflammation model

LPS‐induced astrocyte inflammation model was established to mimic the cascade of inflammation after SCI in vitro.^21^ Astrocytes were cultured from the spinal cord of P1‐P3 mouse.^17^ In brief, the spinal cords were removed, demembranated, chopped, and then incubated with 0.125% trypsin (C0208, Beyotime) at 37°C for 20 min. After mechanical disruption, the cells were seeded on poly‐L‐lysine (ST508, Beyotime)‐coated culture dishes and incubated in DMEM containing 10% fetal bovine serum (FBS, Gibco). After 6–10 days, other glia cells were separated by shaking at 250 rpm for 12 h. The astrocytes were collected and plated into poly‐L‐lysine‐coated dishes or coverslips. For LPS treatment (1 μg/mL, Solarbio) or ALX (10 μM, MCE), astrocytes were starved in DMEM without serum for 24 h before treatment.

### 2.5 cDNA constructs

IRF3‐shRNA plasmid was purchased from Tsingke. The sequence of IRF3‐shRNA1 was 5′‐GCTATTGTTTCTGATCCTTCT‐3′; the sequence of IRF3‐shRNA2 was 5′‐TGCAGCTCCACGAGACCTTTA‐3′. The expression constructs were verified by sequencing.

### 2.6 Western blot

The animals were sacrificed at 1, 3, and 7 days after SCI, and spinal cord tissues were collected. Proteins were extracted with a lysis buffer: ice‐cord RIPA‐buffer (P0013B, Beyotime), 100 mM PMSF, 100 mM PIC, and incubated in a shaker at 4°C for 30 min. The BCA (23,225, Thermo Scientific) method was used for the protein quantitation. The proteins were separated by 8%, 10%, or 12% SDS‐PAGE and transferred to a nitrocellulose membrane by wet transfer. Subsequently, they were blocked with 5% milk and incubated with different primary antibodies for overnight at 4°C. After washing three times with 0.1% Tween 20 in TBST, the blots were incubated with appropriate secondary antibodies (1:10,000, #7074, #7076, CST) for 1 h at room temperature. The primary antibodies included rabbit anti‐YAP (1:1000, #14074, CST), rabbit anti‐phospho‐YAP (1:1000, #4911, CST), rabbit anti‐NF‐κB2 p100/p52 (1:1000, #4882, CST), rabbit anti‐RelB (1:1000, #4922, CST), rabbit anti‐phospho‐IRF3 (1:1000, #37829, CST), mouse anti‐GAPDH (1:5000, #2118, CST), rabbit anti‐Bcl‐2 (1:1000, ab194583, Abcam), rabbit anti‐Bax (1:1000, ab32503, Abcam), rabbit anti‐cleaved‐caspase3 (1:1000, #9661, CST), mouse anti‐GFAP (1:1000, MAB360, Millipore), rabbit anti‐NF‐κB p65 (1:1000, #8242, CST), rabbit anti‐Phospho‐TBK1 (Ser172) (1:1000, #AF8190, Affinity), and rabbit anti‐TBK1 (1:1000, #DF7026, Affinity). The protein bands were detected by the ECL detection kit (BGT‐006, Biomedical Technology) with an imaging system (GV 6000Plus, BLT). The densities of bands were analyzed by ImageJ software.

### 2.7 Nissl staining

After animals were anesthetized and perfusing the mice with 0.1 mol/L PBS followed by 4% PFA, the spinal cord was collected. The spinal cord was fixed in 4% PFA for 24 h, then dehydrated with 30% sucrose and embedded in an optimal cutting temperature compound (#4583, SAKURA). The spinal cord segments containing the lesion were cut into 14‐μm‐thick transverse sections using a freezing microtome (Cryostar NX50 OP, Thermo Scientific). For serial sections, the spinal cord segments containing the lesion were cut in the horizontal plane. Serial sections were collected spanning the cephalad to cauda axis. Every three sections were stained for Nissl stain and the number quantified. The sample size in each group was from four mice. The sections were subsequently dehydrated, incubated with 0.1% cresyl violet for 3 min at room temperature, and then rinsed in double‐distilled water. After that, it was differentiated with 95% alcohol, conventionally dehydrated, cleared, and covered with neutral resin. Then, the sections were observed with a microscope (VS200, Olympus, Germany). The ventral normal motor neurons and apoptotic neurons were analyzed with ImageJ software.

### 2.8 Hematoxylin and eosin (HE) staining

The frozen tissues were cut into 14‐μm‐thick horizontal sections using a freezing microtome (Cryostar NX50 OP, Thermo Scientific). The spinal cord horizontal sections were stained with hematoxylin and eosin (G1120, Solarbio) to evaluate inflammatory infiltrates around the injured area. After staining with hematoxylin for 1 min, the sections were rinsed with water for 120 s. Then, the sections were incubated in 1% acidic liquid alcohol for 30 s and rinsed with water for 60 s. The sections were stained with eosine for 1 min, followed by dehydration with graded alcohol, and cleared in xylene. Finally, the sections were mounted by neutral resins for further analysis.

### 2.9 Immunofluorescent staining

After perfusing with 0.1 mol/L PBS followed by 4% PFA, the mice spinal cord samples were fixed in 4% PFA for 24 h, then dehydrated with 30% sucrose until they sank. The samples were cut into 14‐μm‐thick transverse sections with a freezing microtome. Then, the sections were fixed with 4% PFA for 30 min, and the antigens repaired for 30 min at 90°C using sodium citrate antigen retrieval solution (C1032, Solarbio). After treated with 0.3% Triton X‐100 (T8200, Solarbio) for 30 min, the sections were blocked with 5% BSA plus 0.3% Triton X‐100 for 1 h and incubated with primary antibodies for overnight at 4°C. They were subsequently washed thrice with PBS and incubated for 1 h at room temperature with the appropriate secondary antibodies (1:1000, Invitrogen). The primary antibodies included rabbit anti‐YAP (1:500, #14074, CST), mouse anti‐GFAP (1:1000, MAB360, Millipore), goat anti‐Iba1 (1:500, ab5076, Abcam), rabbit anti‐NeuN (1:1000, MAB377, Millipore), rabbit anti‐Ki67 (1:500, AB9260, Millipore), rabbit anti‐NF‐κB2 p100/p52 (1:1000, #4882, CST), rabbit anti‐p‐IRF3 (1:1000, #37829, CST), rabbit anti‐Cleaved‐caspase3 (1:100, #9661, CST), rabbit anti‐Phospho‐TBK1 (Ser172) (1:100, #AF8190, Affinity), rabbit anti‐TBK1 (1:100, #DF7026, Affinity), mouse anti‐F4/80 (1:500, ab664, Abcam), and mouse anti‐Iba1 (1:100, GB12105, Servicebio). Meanwhile, the nucleus was marked with DAPI (1:1000, #4083, CST). Images were captured using a confocal laser scanning microscope (FV3000RS, Olympus) or a fluorescence microscope (VS200, Olympus).

For cultured cell staining, the cells were washed thrice with PBS and fixed in 4% PFA for 30 min. After treated with 0.1% Triton X‐100 for 15 min, the sections were blocked with 5% BSA for 1 h and incubated with primary antibodies for overnight at 4°C. Subsequently, the cells were washed thrice with PBS and incubated for 1 h at room temperature with the appropriate secondary antibodies (1:1000, Invitrogen). Meanwhile, DAPI (1:1000, #4083, CST) was stained for nuclear. The primary antibodies included rabbit anti‐YAP (1:500, #14074, CST), mouse anti‐GFAP (1:1000, MAB360, Millipore), rabbit anti‐Ki67 (1:500, AB9260, Millipore), rabbit anti‐p‐IRF3 (1:500, #37829, CST), mouse anti‐ALDH1L1 (1:1000, ab56777, Abcam), rabbit anti‐ALDH1L1 (1:1000, ab177463, Abcam), and mouse anti‐PH3 (1:2000, ab14955, Abcam). Images were captured using a confocal laser scanning microscope (FV3000RS, Olympus) or a fluorescence microscope (VS200, Olympus).

### 2.10 qRT‐PCR

Total RNA was extracted from the spinal cords after SCI (3 and 7 days) or astrocytes using RNA‐easy isolation reagent (R701‐01‐AA, Vazyme) following according to manufacturer's instructions. The RNA was reverse transcribed into cDNA using HiScript III RT SuperMix (R323‐01, Vazyme). The expression levels of IL‐1β, IL‐6, TNF‐α, TBK1, and β‐actin were quantified using the ChamQ Universal SYBR qPCR Master Mix (Q711‐02, Vazyme) on the real‐time PCR detection system (Bio‐Rad). The samples were amplified independently at least three times. Relative gene expression was converted using the 2^−∆∆Ct^ method. The primer used included the following:

IL‐1β: F, 5′‐GCAACTGTTCCTGAACTCAACT‐3′;
R, 5′‐ATCTTTTGGGGTCCGTCAACT‐3′.
IL‐6: F, 5′‐CCAAGAGGTGAGTGCTTCCC‐3′;
R, 5′‐CTGTTGTTCAGACTCTCTCCCT‐3′.
TNF‐α: F, 5′‐CCCTCACACTCAGATCATCTTCT‐3′;
R, 5′‐GCTACGACGTGGGCTACAG −3′.
β‐actin: F, 5′‐GGCTGTATTCCCCTCCATCG‐3′;
R, 5′‐CCAGTTGGTAACAATGCCATGT −3′.

### 2.11 Image analysis

Images were captured using a confocal laser scanning microscope (FV3000RS, Olympus) or a fluorescence microscope (VS200, Olympus). Brightness and contrast of the images were adjusted, pseudo‐colors was applied for presentation, and images were cropped with ImageJ (NIH). When imaging was taken for quantification, image capture and processing were kept constant. 3–4 slides across the dorsal‐ventral axis were imaged per spinal cord, and the average value was taken for quantitative analysis. At least three mice were used for each group. All images of the same experiment were taken under exactly the same conditions. The images were further processed using professional Olyvia software (media cybernetics, Rockville, USA) and ImageJ. Data were presented as related positive cell numbers around the injury site in spinal cord or the mean value of integrated optical density with an average of 1 mm^2^.

### 2.12 Statistical analysis

Each experiment was performed at least three times, and the data were presented as mean ± SEM and were analyzed with GraphPad software (Graph Pad Software, Inc., San Diego, CA). D'Agostino‐Pearson omnibus normality test was used to assess data distribution. All data were subject to tests for normality. Comparisions between two groups were made with unpaired two‐tailed Student's *t*‐test. Multiple group comparisions were used one‐way ANOVA followed by Tukey's post hoc test or two‐way ANOVA followed by Bonferroni's post hoc test for statistical analysis. *p* ˂ 0.05 was considered statistically significantly.

## 3 RESULTS

### 3.1 TBK1/IRF3 and noncanonical NF‐κB signaling were upregulated in astrocytes and neurons after SCI

**FIGURE 1:**
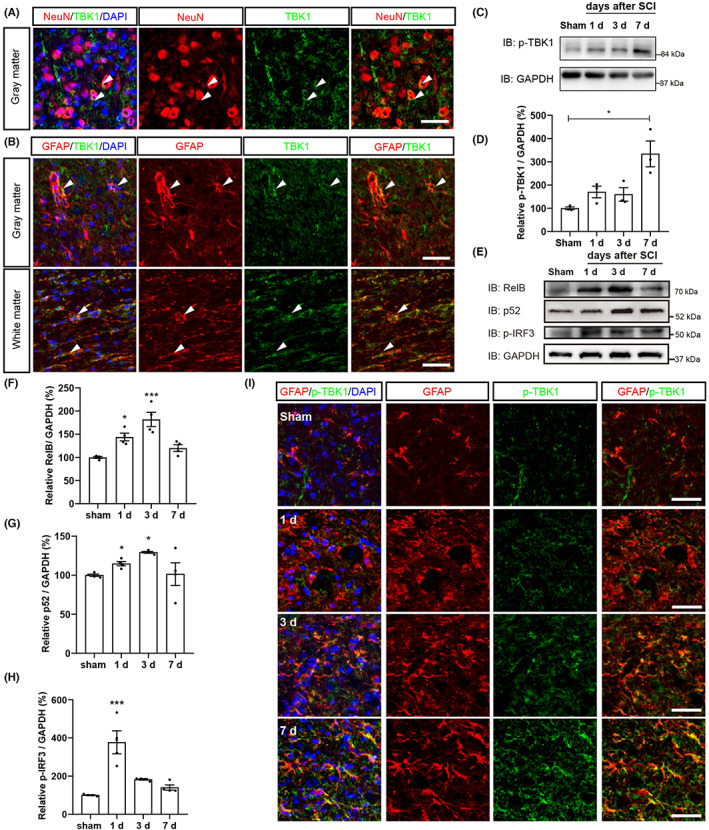
The expression of TBK1/IRF3 and noncanonical NF‐κB signaling pathway proteins in the spinal cord after SCI. (A, B) Double immunostaining analysis of TBK1(green) and NeuN (red) (A), TBK1(green), and GFAP (red) (B) in the spinal cord gray and white matter. (C) Western blot detected the expression of p‐TBK1 protein in spinal cords after SCI. (D) Quantitative analysis of the relative p‐TBK1 levels as shown in (C) (*n* = 4, per group). (E) Western blot detected the expression of RelB, p‐IRF3, and p52 protein in spinal cords after SCI. (F–H) Quantitative analysis of the relative RelB (F), p52 (G), and p‐IRF3 (H) levels as shown in (E) (*n* = 4, per group, normalized to sham group). (I) Immunostaining analysis of p‐TBK1 (green) and GFAP (red) 500 μm away from the core of the lesion site after SCI. Quantitative data were analyzed using one‐way ANOVA with Tukey tests. D'Agostino‐Pearson omnibus normality test was used to assess data distribution. All data were subject to tests for normality. Scale bars, 20 μm. Data were presented as the mean ± SEM. *p ˂ 0.05, ***p ˂ 0.001, compared with sham group.

To explore the functions of TBK1 and downstream signaling pathway in regulating innate immune responses after SCI, we first detected the temporal and spatial expression pattern of TBK1 and its activated form, phosphorylated TBK1 (p‐TBK1), in the spinal cords. As shown in Figure 1A, immunofluorescence staining showed that TBK1 was detected in NeuN^+^ cells in the spinal cord gray matter. In addition, TBK1 protein was also detected in GFAP^+^ astrocytes and Iba1^+^ microglia in the spinal cord gray and white matter (Figure 1B, Figure S4A). We performed western blotting to examine the total expression of TBK1 in the lesion area from day 0 to day 7 and found that there was no significant upregulation of TBK1 at lesion site after SCI (Figure S4B,C). TBK1 Ser172 is phosphorylated in its activation loop, which is important for its kinase activity, including downstream phosphorylation of IRF3.^22^, ^23^ Next, the expression of p‐TBK1 and the downstream molecules were examined by western blot analysis from day 0 to day 7 after SCI. As shown in Figure 1C, the expression of p‐TBK1 protein was significantly upregulated at 7 days after SCI (Figure 1C,D). Meanwhile, we also detected the increased expression of p‐TBK1 in GFAP^+^ astrocytes at 7 days after SCI (Figure 1I). Additionally, western blot showed that the expression of p52 and RelB proteins was significantly upregulated at lesion site from 1 d and with a peak at 3 days after SCI, and the expression of p‐IRF3 protein was increased and peaked at 1 day after SCI (Figure 1E–H). As shown in Figure S1A–D, we detected the expression of p52 and p‐IRF3 in GFAP^+^ and NeuN^+^ cells in the sham group. Together, these results suggested that TBK1 and its downstream signaling pathway, such as TBK1/IRF3 and noncanonical NF‐κB signaling pathway, were activated in spinal cord after SCI, indicating that TBK1‐medicated innate immune responses may play an important role in the pathophysiology of spinal cord injury.

### 3.2 The behavioral functional recovery of SCI mice was improved by the inhibition of TBK1

**FIGURE 2:**
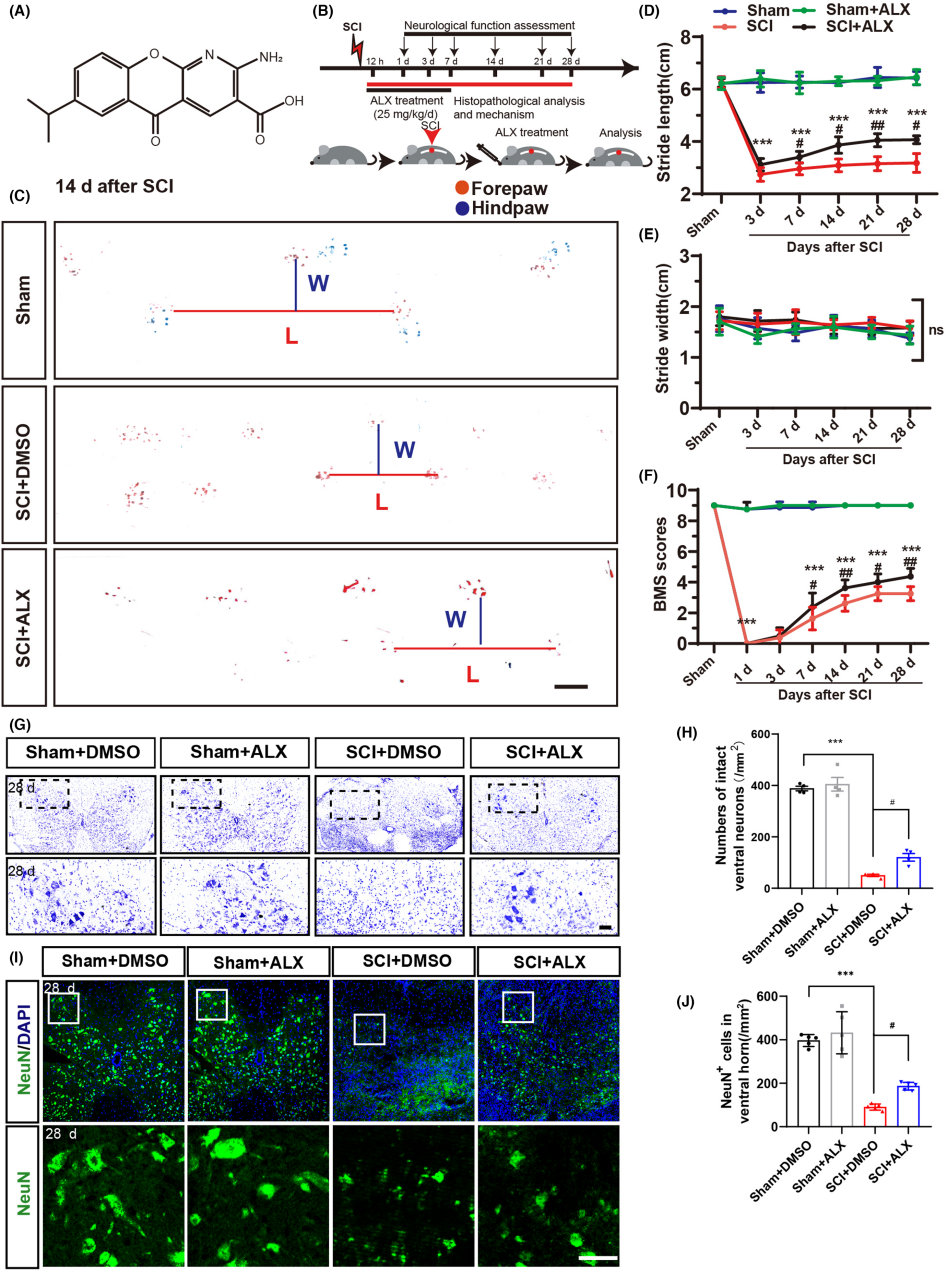
The behavioral functional recovery of SCI mice was improved by inhibition of TBK1. (A) The chemical structure of ALX. (B) Experimental scheme of ALX treatment after SCI. (C) Representative footprint images of Sham, SCI + DMSO, and SCI + ALX groups at 14 days after SCI. Scale bars, 10 mm. (D, E) Quantitative footprint analysis of stride length (D) and stride width (E) in different group over a 28 period after SCI (*n* = 6, per group). (F) Quantitative BMS scoring analysis of hindlimb locomotor performance at different stages after SCI (*n* = 8, per group). (G) Typical Nissl staining images of intact ventral neurons in the spinal cord at 28 days after SCI. (H) Quantitative analysis of density of ventral motor neurons as shown in (G) (*n* = 4, per group). (I) Immunostaining analysis of NeuN in the spinal cord at 28 days after SCI. (J) Quantitative analysis of density of NeuN^+^ cells as shown in (I) (*n* = 5, per group). (D–F) Quantitative data were analyzed using two‐way ANOVA (repeated measures) with Bonferroni tests. (H, J) Quantitative data were analyzed using one‐way ANOVA with Tukey tests. D'Agostino‐Pearson omnibus normality test was used to assess data distribution. All data were subject to tests for normality. Scale bars, 50 μm. Data were presented as the mean ± SEM. ***p ˂ 0.001, compared with Sham group; ^#^ *p* ˂ 0.05, ^##^ *p* ˂ 0.01, ^###^ *p* ˂ 0.001, compared with SCI + DMSO group.

To study the function of TBK1 signaling in spinal cord injury, ALX as a specific small molecule inhibitor of TBK1was chosen to treat SCI mice (Figure 2A). Indeed, compared with the vehicle group, the expression of p‐TBK1 was significantly decreased in the SCI + ALX group at 7 days after SCI (Figure S2A,B). Interestingly, the fluorescence intensity of p‐TBK1 in reactive astrocyte was decreased in the SCI + ALX group at 7 days after SCI, compared with the vehicle group (Figure S2C). After the mice woke up after surgery, the treatment group was given ALX at a dose of 25 mg/kg/day by gavage (Figure 2B). The footprint analysis and BMS scores assays were used to evaluate the effects of locomotion recovery following a SCI. Interestingly, the stride length in footprint assays was significantly improved in mice treated with ALX from day 7 to day 28 after SCI (Figure 2C,D). However, there was no significant difference in stride width between control‐treated mice and ALX‐treated mice after SCI (Figure 2E). Furthermore, BMS scores showed that ALX treatment significantly improved the locomotion function at 7, 14, 21, and 28 days after SCI (Figure 2F). These results suggest that inhibition of TBK1 activity by ALX has a therapeutic effect on SCI. To further explore whether the protective effects of ALX on SCI were due to decrease of the loss of motor neurons, Nissl staining and immunostaining on transverse sections of spinal cord tissue were performed after SCI. Nissl staining showed that the loss of intact ventral motor neurons were significantly increased at 28 days after SCI; however ALX treatment significantly inhibited the loss of intact ventral motor neurons (Figure 2G,H). Furthermore, immunostaining showed the dramatic reduction of NeuN^+^ neurons at 28 days after SCI. ALX treatment significantly decreased the loss of NeuN^+^ neurons (Figure 2I,J). Together, these results indicate that inhibition of TBK1 activity alleviates the loss of ventral motor neurons after SCI, which is responsible for the improvement of functional recovery.

### 3.3 Neuroinflammation at SCI early phase was alleviated by inhibition of TBK1 activity

**FIGURE 3:**
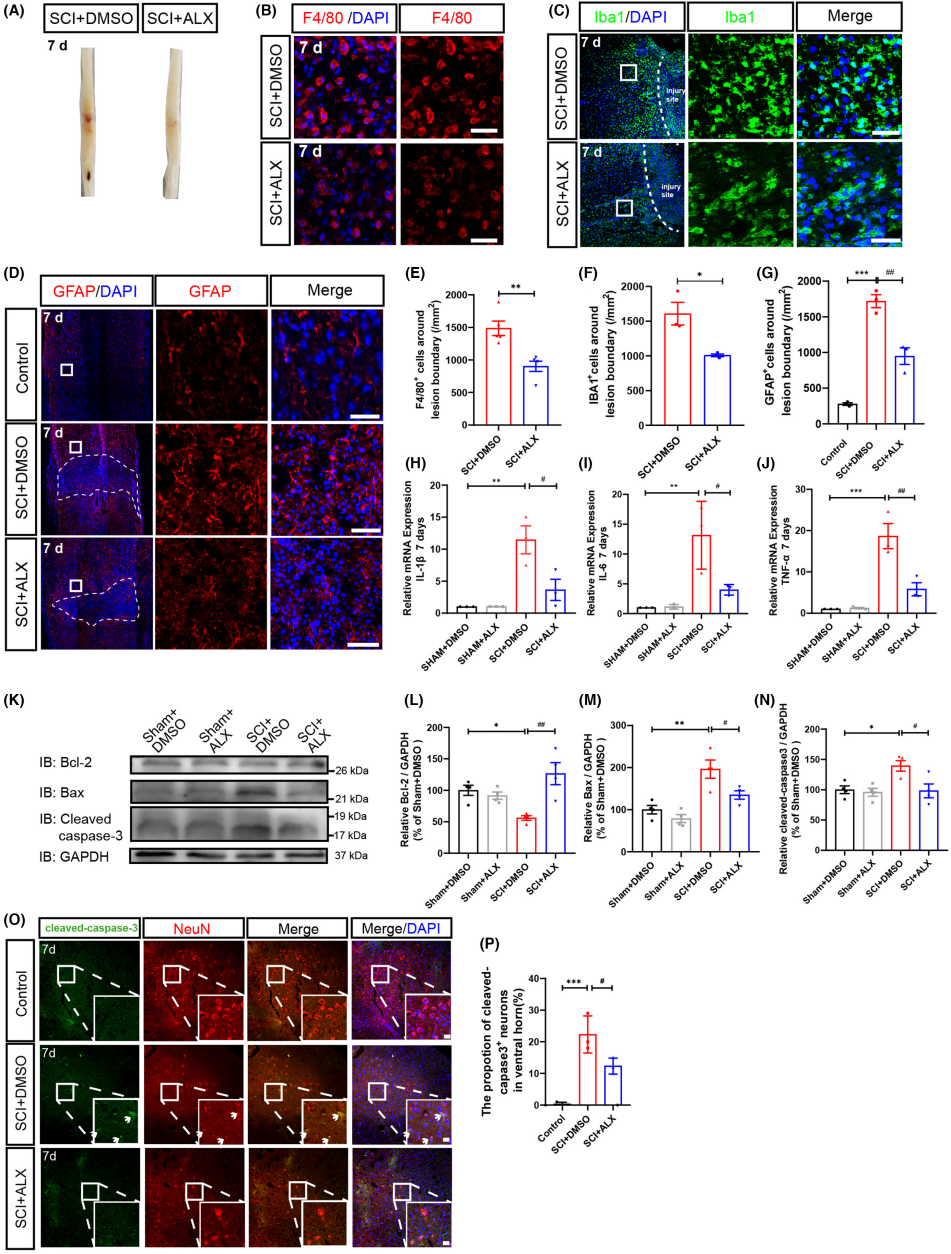
Neuroinflammation at SCI early phase was alleviated by the inhibition of TBK1 activity. (A) The typical images of hematoma in spinal cords at 7 days after SCI. (B) Immunostaining analysis of F4/80 (red) 500 μm away from the core of the lesion site at 7 days after SCI. Scale bars, 20 μm. (C, D) Immunostaining analysis of Iba1 (green, C) and GFAP (red, D) 500 μm away from the core of the lesion site at 7 days after SCI. Scale bars, 50 μm. (E–G) Quantitative analysis of the density of F4/80^+^ (B, *n* = 5 per group), Iba1^+^ (C, *n* = 3 per group), and GFAP^+^ (D, *n* = 3 per group). (H–J) Relative mRNA expression of inflammatory cytokines, such as IL‐1β (H), IL‐6 (I), and TNF‐α (J) at 7 days after SCI (*n* = 3, per group). (K) Western blot detected Bcl‐2, Bax, and cleaved‐caspase3 expression at 7 days after SCI. (L–N) Quantitative analysis of relative Bcl‐2 (L), Bax (M), cleaved‐caspase3 (N) protein levels as shown in (K) (*n* = 4, per group). (O) Immunostaining analysis of cleaved‐caspase3 (green) and NeuN (red) cells at 7 days after SCI. Scale bars, 20 μm. (P) Quantitative analysis of percentages of cleaved‐caspase3^+^ neurons as shown in (O) (*n* = 4, per group). D'Agostino‐Pearson omnibus normality test was used to assess data distribution. All data were subject to tests for normality. Quantitative data were analyzed using one‐way ANOVA with Tukey tests and Student's unpaired two‐tailed *t* test. Data were presented as the mean ± SEM. *p ˂ 0.05, **p ˂ 0.01, ***p ˂ 0.001, compared with Sham + DMSO group; ^#^ *p* ˂ 0.05, ^##^ *p* ˂ 0.01, compared with SCI + DMSO group.

It has been reported that a cascade of destructive events, such as oxidative stress, hematoma, ischemia, and inflammatory events in the injured area reflected the severity of secondary injury after SCI.^3^, ^24^ As shown in Figure 3A, compared with the vehicle group, the hematoma was decreased in the SCI + ALX group at 7 days after SCI. As the main immune cells in the spinal cord, macrophages, astrocytes, and microglia were closely related to the neuroinflammatory response after SCI. F4/80, a cell surface glycoprotein, was expressed in a variety of mature macrophages.^25^ As shown in Figure 3B, immunostaining showed that the infiltration of F4/80^+^ cells was significantly reduced in ALX‐treated mice at 7 days after SCI. Meanwhile, we found that Iba1^+^ cells were also significantly decreased around the injury site in ALX‐treated mice (Figure 3C,F). Further double immunostaining showed that the number of GFAP^+^ astrocytes were significantly decreased in ALX‐treated mice at 7 days after SCI (Figure 3D,G), compared with that in control‐treated mice. We next examined whether inhibition of TBK1 activity affected neuroinflammation and the release of inflammatory cytokines such as interleukin‐1β (IL‐1β), interleukin‐6 (IL‐6), and tumor necrosis factor alpha (TNF‐α) after SCI. As expected, qPCR showed that the IL‐1β, IL‐6, and TNF‐α mRNA were significantly upregulated in spinal cords at 7 days after SCI; however, ALX treatment significantly inhibited the increase of IL‐1β, IL‐6, and TNF‐α mRNA (Figure 3H–J). Since apoptosis plays a critical role in the secondary injury of SCI, we next examined whether the inhibition of TBK1 activity protected neurons against SCI‐induced apoptosis. Indeed, the expression of apoptotic factors such as Bax and cleaved caspase‐3 was significantly upregulated in spinal cords at 7 days after SCI; however, ALX treatment significantly inhibited the increase of Bax and cleaved caspase‐3, compared with that in control‐treated mice. Meanwhile, the expression of antiapoptotic factor Bcl‐2 was significantly decreased in spinal cords at 7 days after SCI; however, ALX treatment significantly upregulated the expression of Bcl‐2 (Figure 3K–N). Moreover, double immunostaining showed that the percentage of cleaved caspase‐3‐positive neurons was significantly decreased in SCI mice treated with ALX (Figure 3O,P). Taken together, these results suggest that inhibition of TBK1 activity alleviates the neuroinflammation in spinal cords at SCI early phase and protects neurons against SCI‐induced apoptosis.

### 3.4 Inhibition of TBK1 activity reduced the proliferation of reactive astrocytes in a mice model of SCI and in a model of astrocyte inflammation in vitro

**FIGURE 4:**
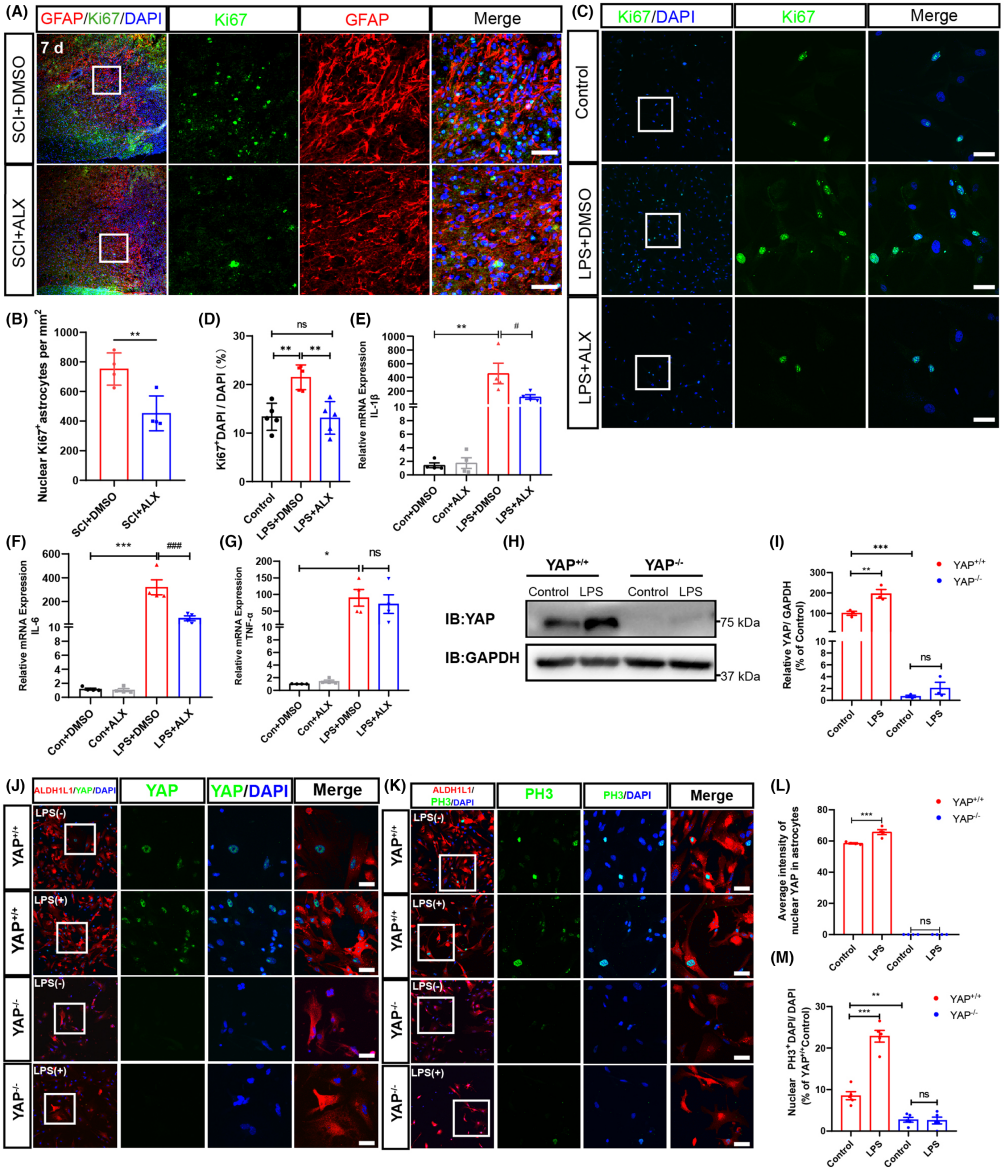
Inhibition of TBK1 activity reduced the proliferation of reactive astrocytes in a mice model of SCI and in a model of astrocyte inflammation in vitro. (A) Double immunostaining analysis of Ki67 (green) and GFAP (red) around the lesion site in the spinal cord at 7 days after SCI. Scale bars, 50 μm. (B) Quantitative analysis of the number of nuclear Ki67^+^ cells in GFAP^+^ astrocytes as shown in (A) (*n* = 4, per group). (C) Immunostaining analysis of Ki67 (green) in LPS‐induced astrocyte inflammation model. Scale bars, 20 μm. (D) Quantitative analysis of the percentages of Ki67^+^ cells in total observed astrocytes as shown in (C) (*n* = 4, per group). (E–G) Relative mRNA expression of inflammatory cytokines, such as IL‐1β (E), IL‐6 (F), and TNF‐α (G) in vitro (*n* = 4, per group). (H) Western blot detected YAP protein expression in primary cultured astrocytes from *yap* ^*GFAP*^‐CKO mice and *yap* ^*f/f*^ mice. (I) Quantitative analysis of the relative YAP level as shown in (H) (*n* = 3, per group). (J) Double immunostaining analysis of YAP (green) and ALDH1L1 (red) in primary cultured astrocytes from control and *yap*‐deficient mice without or with LPS treatment (1 μg/mL). Scale bars, 50 μm. (K) Double immunostaining analysis of PH3 (green) and ALDH1L1 (red) in primary cultured astrocytes from control and *yap*‐deficient mice without or with LPS treatment (1 μg/mL). (L) Quantitative analysis of the average intensity of nuclear YAP as shown in (J) (*n* = 4 fields each group). Scale bars, 50 μm. (M) Quantitative analysis of PH3^+^ DAPI/DAPI as shown in (K) (*n* = 5 fields each group). D'Agostino‐Pearson omnibus normality test was used to assess data distribution. All data were subject to tests for normality. Quantitative data were analyzed using one‐way ANOVA with Tukey tests and Student's unpaired two‐tailed *t* test. Data were presented as the mean ± SEM. *p ˂ 0.05, **p ˂ 0.01, ***p ˂ 0.001, compared with control group; ^#^ *p* ˂ 0.05, ^###^ *p* ˂ 0.001, compared with LPS + DMSO group.

We next examined whether the reduction of GFAP^+^ astrocytes was due to the decrease of proliferation of astrocytes by the inhibition of TBK1 activity. As excepted, as shown in Figure 4A, the number of Ki67^+^/GFAP^+^ cells around the lesion site was significantly decreased in ALX‐treated mice, compared with that in control‐treated mice (Figure 4A,B). To further confirm the effects of ALX on astrocytes, lipopolysaccharide (LPS)‐induced astrocyte proliferation and neuroinflammation model were established. First, we performed the western blotting to examine the expression of p‐TBK1 in LPS‐induced astrocyte inflammation model. Compared with the LPS + DMSO group, the expression of p‐TBK1 was significantly decreased in the LPS + ALX group (Figure S3A,B). In addition, we performed the costaining of GFAP and p‐TBK1 to examine whether ALX treatment affected the TBK1 expression of reactive astrocyte (Figure S3C). As shown in Figure S3D, the fluorescence intensity of p‐TBK1 in GFAP^+^ astrocyte was significantly decreased in the LPS + ALX group, compared with the LPS + DMSO group. Indeed, the proliferation of reactive astrocytes was significantly increased by LPS treatment. However, LPS failed to induce the proliferation of ALX‐treated astrocytes (Figure 4C,D), indicating that TBK1 was required for the proliferation of astrocytes induced by LPS. Additionally, qPCR showed that the IL‐1β, IL‐6, and TNF‐α mRNA were significantly upregulated in astrocytes after LPS treatment; however, ALX treatment inhibited the increase of IL‐1β and IL‐6 mRNA in astrocytes induced by LPS (Figure 4E,F). Interestingly, we found that there was no significant difference in the level of TNF‐α mRNA after ALX treatment (Figure 4G). Since our previous studies have shown that YAP is required for the proliferation and the activation of astrocytes after SCI,^17^ we next examined whether YAP signaling as a downstream of TBK1 was required for the astrocytic proliferation after SCI. Then, we isolated the spinal cord tissue of P1‐P3 *yap* ^*GFAP*^‐CKO conditional KO mice and *yap* ^*f/f*^ mice for primary astrocyte culture. As excepted, as shown in Figure 4H–J, YAP was deleted in astrocytes. Interestingly, LPS failed to induce the proliferation of YAP deletion astrocyte (Figure 4K,M). Together, these results suggest that ALX inhibits the proliferation of reactive astrocytes in vivo and in vitro and alleviates the neuroinflammation response of astrocytes in vitro. In addition, LPS may promote the reactive astrocytic proliferation though controlling nuclear translation of YAP.

### 3.5 YAP signaling as a downstream of TBK1 was required for the reactive astrocytic proliferation after SCI

**FIGURE 5:**
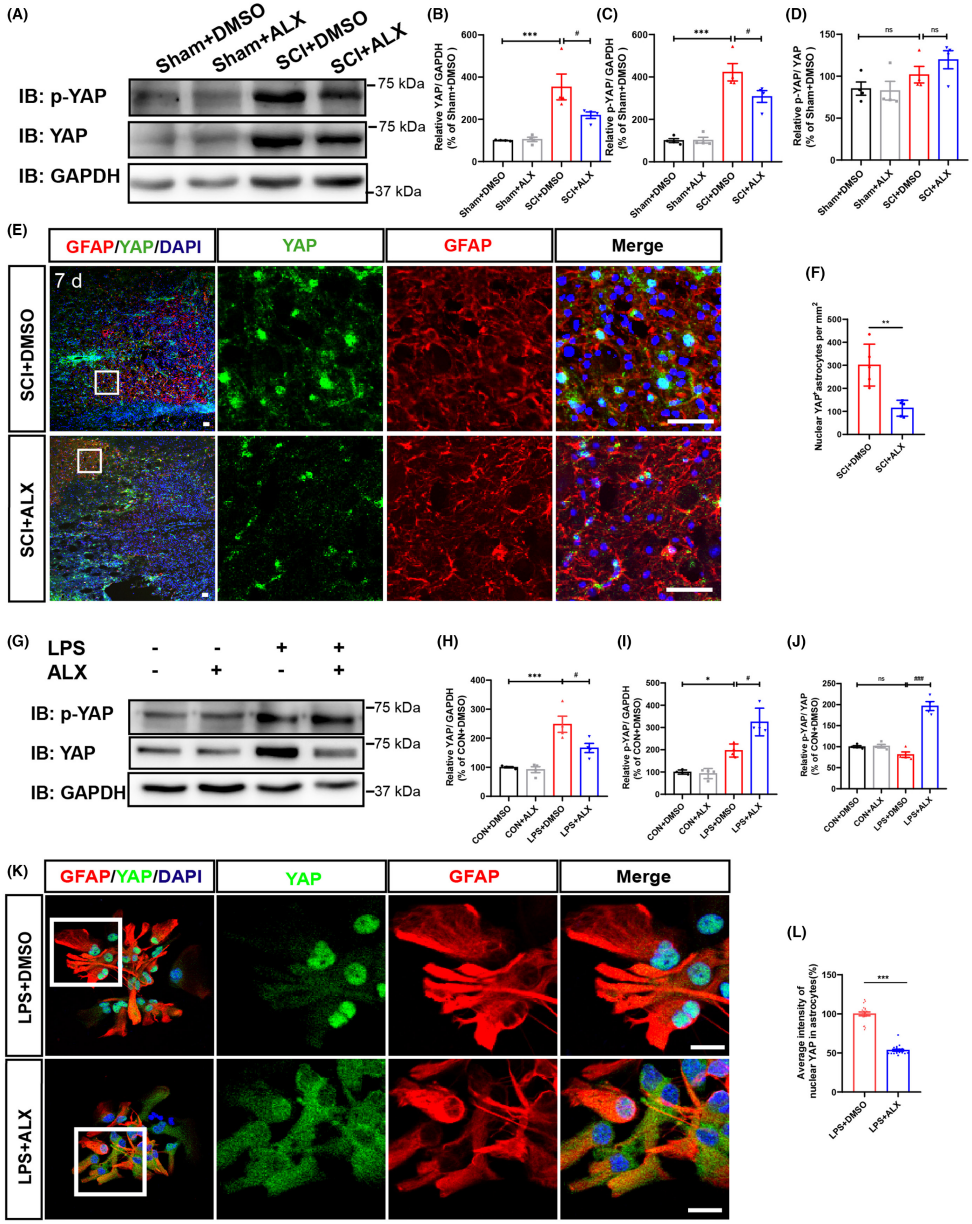
YAP signaling as a downstream of TBK1 was required for the reactive astrocytic proliferation after SCI. (A) Western blot detected YAP and p‐YAP expression in the lesion site at 7 days after SCI. (B–D) Quantitative analysis of the relative YAP (B), p‐YAP (C), and p‐YAP/YAP (D) levels (*n* = 4, per group). (E) Double immunostaining analysis of YAP (green) and GFAP (red) around the lesion site in the spinal cord at 7 days after SCI. ALX treatment effectively inhibits the nuclear translocation of YAP in vivo. Scale bars, 50 μm. (F) Quantitative analysis of nuclear YAP^+^ cells number in GFAP^+^ astrocytes as shown in (E) (*n* = 4, per group). (G) Western blot detected YAP and p‐YAP expression in LPS‐induced astrocyte‐mediated inflammation response model with or without ALX treatment. (H–J) Quantitative analysis of YAP (H), p‐YAP (I) and the relative p‐YAP/YAP (J) (*n* = 4, per group). (K) Immunostaining analysis of YAP (green) and GFAP (red) in LPS‐induced astrocyte inflammation model with or without ALX treatment. ALX treatment effectively inhibit the nuclear translocation of YAP in vitro. Scale bars, 20 μm. (L) Quantitative analysis of the average intensity of nuclear YAP as shown in (K) (*n* = 4, per group). (B–D, H–J) Quantitative data were analyzed using one‐way ANOVA with Tukey tests. (F, L) Quantitative data were analyzed using Student's unpaired two‐tailed *t* test. D'Agostino‐Pearson omnibus normality test was used to assess data distribution. All data were subject to tests for normality. Data were presented as the mean ± SEM. *p ˂ 0.05, **p ˂ 0.01, ***p ˂ 0.001, compared with control group; ^#^ *p* ˂ 0.05, ^##^ *p* ˂ 0.01, ^###^ *p* ˂ 0.01, compared with LPS + DMSO group or SCI + DMSO group.

As shown in Figure 5A–D, the expression of YAP protein was significantly upregulated in spinal cords at 7 days after SCI; however, the expression of YAP was significantly downregulated in spinal cords of ALX‐treated mice, whereas the relative ratio of phospho‐YAP (p‐YAP)/YAP did not change significantly. Moreover, the number of nuclear YAP^+^ astrocytes was significantly decreased at the lesion site in ALX‐treated mice at 7 days after SCI, compared with that in control‐treated mice (Figure 5E,F). Furthermore, western blot results showed that ALX treatment significantly inhibited the increase of YAP in LPS‐induced astrocytes, whereas ALX treatment significantly increased the relative ratio of phospho‐YAP (p‐YAP)/YAP (Figure 5G–J). Similarly, immunostaining confirmed that ALX treatment inhibited the increase of the intensity of nuclear YAP in LPS‐induced astrocytes (Figure 5K,L). Taken together, these results suggest that treatment with ALX can effectively inhibit the nuclear translocation of YAP in vivo spinal cord injury models and in vitro astrocyte LPS‐stimulated models. YAP signaling as a downstream of TBK1 may be required for the astrocytic proliferation after SCI, and ALX exerts the neuroprotective effects and inhibits astrocyte‐mediated inflammatory response through inhibiting YAP after SCI.

### 3.6 Inhibition of TBK1 activity promoted the activation of the noncanonical NF‐κB signaling pathway and inhibited p‐IRF3 in LPS‐induced astrocytes

**FIGURE 6:**
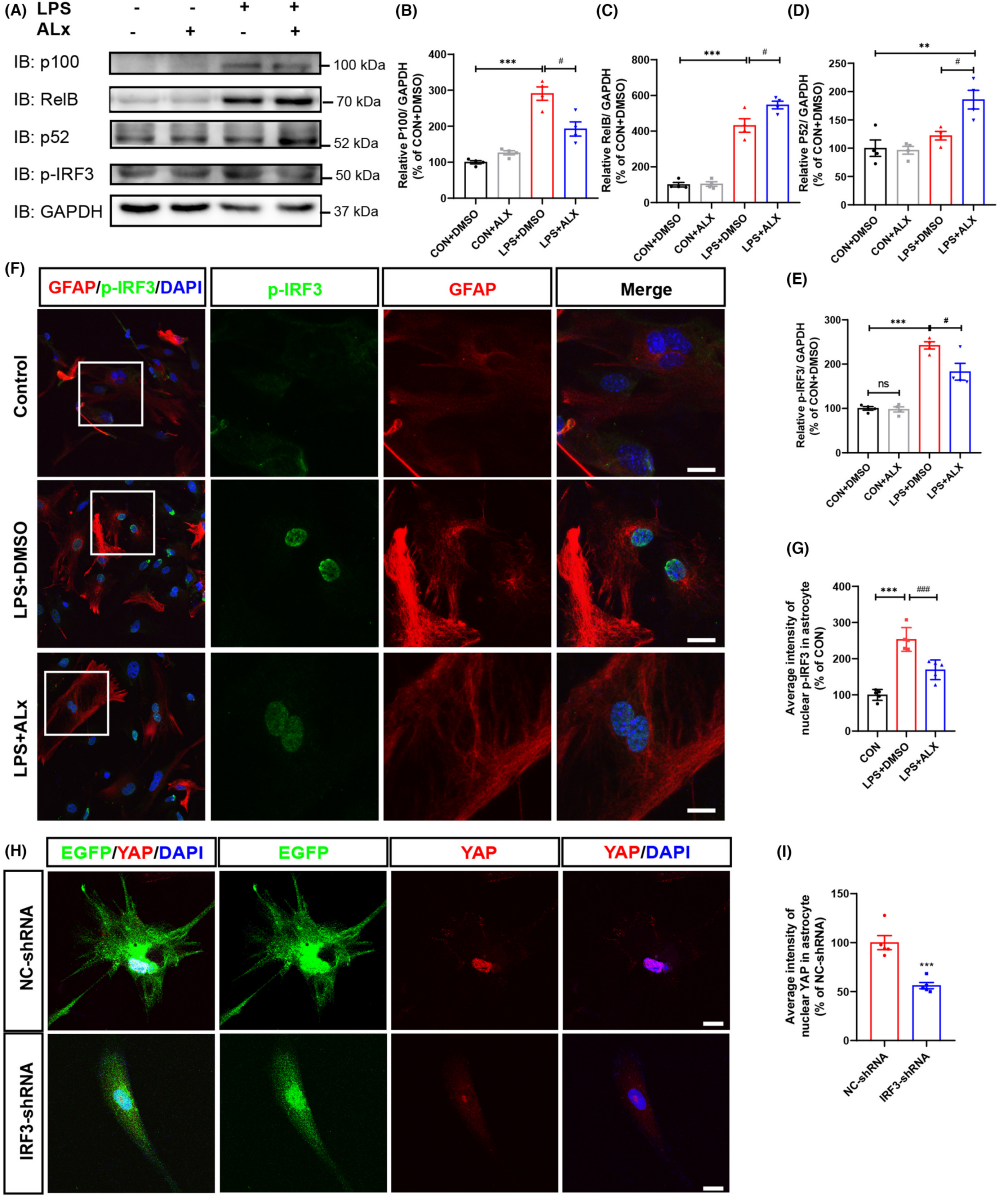
Inhibition of TBK1 activity promoted the activation of the noncanonical NF‐κB signaling pathway and inhibited pIRF3 in LPS‐induced astrocytes model. (A) Western blot detected p100, RelB, p52, and p‐IRF3 proteins expression in LPS‐induced astrocyte‐mediated inflammation response model. (B–E) Quantitative analysis of the relative p100 (B), RelB (C), p52 (D), and p‐IRF3 (E) levels as shown in (A) (*n* = 4, per group). (F) Double immunostaining analysis of p‐IRF3 (green) and GFAP (red) in LPS‐induced astrocyte inflammation model. Scale bars, 20 μm. (G) Quantitative analysis of the average intensity of nuclear p‐IRF3 as shown in (F) (*n* = 4, per group). (H) Immunostaining analysis of YAP (red) and EGFP (green) in LPS‐induced astrocyte inflammation model after transfection with IRF3‐shRNA and control‐shRNA. Scale bars, 20 μm. (I) Quantitative analysis of the average intensity of nuclear YAP as shown in (H) (*n* = 4, per group). (B–E) Quantitative data were analyzed using one‐way ANOVA with Tukey tests. (G, I) Quantitative data were analyzed using Student's unpaired two‐tailed *t* test. D'Agostino‐Pearson omnibus normality test was used to assess data distribution. All data were subject to tests for normality. Data were presented as the mean ± SEM. **p ˂ 0.01, ***p ˂ 0.001, compared with the CON + DMSO group. ^#^ *p* ˂ 0.05, ^###^ *p* ˂ 0.001, compared with LPS + DMSO group.

To investigate the underlying mechanism of TBK1 in regulating reactive astrogliosis, noncanonical NF‐κB signaling pathway‐related proteins, such as p100, p52 and RelB, was first examined in LPS‐induced astrocytic activation model. As expected, the expression of p100, p52, and RelB was significantly upregulated in astrocytes treated with LPS after 3 h (Figure 6A–D), which indicate that LPS treatment induced the activation of noncanonical NF‐κB signaling pathway in astrocytes. Interestingly, ALX treatment significantly downregulated the protein level of p100 in these LPS‐induced astrocytes and upregulated the p52 and RelB proteins (Figure 6A–D). These results indicate that the inhibitory effect of ALX on the astrocytic activation may be mediated by the nonclassical NF‐κB signaling pathway. In addition, we further tested p‐IRF3 protein expression in this model. Interestingly, we found that p‐IRF3 protein expression level was significantly increased in LPS‐treated astrocytes; however, ALX treatment inhibited the increase of p‐IRF3 in LPS‐induced astrocytes (Figure 6A,E). Immunostaining further confirmed the inhibitive effects of ALX on the increase of p‐IRF3 in LPS‐induced astrocytes (Figure 6F,G). To further confirm the TBK1/IRF3 signal regulating YAP activity, the IRF3‐shRNA plasmid was constructed. After transfection with IRF3‐shRNA for 48 h, immunostaining of YAP was performed in astrocytes‐medicate inflammation model. Interestingly, knockdown of IRF3 apparently diminished the nuclear translation of YAP in astrocytes (Figure 6H,I). Taken together, these results suggest that inhibition of TBK1 activity promotes the activation of the noncanonical NF‐κB signaling pathway and inhibits LPS‐induced nuclear translocation of YAP in astrocytes by alleviating phosphorylation of IRF3.

## 4 DISCUSSION

In this study, we show the temporal and spatial expression of TBK1‐medicated innate immune responses proteins after SCI for the first time and propose its working mechanism. Inhibition of TBK1 can promote the activation of noncanonical NF‐κB signaling pathways. Our data demonstrate that the expression of phosphorylation of IRF3 plays an important role in regulating YAP nuclear translocation in LPS‐induced astrocyte inflammation model. Collectively, our study supports the view that ALX is a potential therapeutic drug for SCI targeting TBK1‐medicated innate immune responses, such as noncanonical NF‐κB pathway and TBK1/IRF3 pathway.

Although noncanonical NF‐κB signaling in the CNS has been normally associated with antiviral immune responses,^9^ our data indicated that injury to the spinal cord and LPS‐stimulated astrocytes in culture elevated the protein levels of noncanonical NF‐κB signaling, p100 and RelB. However, previous studies have shown that the expression of p52 and RelB proteins in neurons and oligodendrocytes did not change significantly after SCI.^26^ This may imply that p52 and RelB in other glial cells are involved in the innate immune response after SCI. In our study, we also found that the expression of p100 and RelB proteins was increased in LPS‐stimulated astrocytes in vitro (Figure 6A–C). As a member of the NF‐κB family of transcription factors, RelB is involved in the regulation of various CNS diseases, especially in the neuroinflammatory.^27^, ^28^ Under physiological conditions, RelB is expressed at low levels in primary cultured astrocytes. We found that its expression level rose rapidly after stimulation with LPS (Figure 6A,C). This may be related to the adaptive responses of astrocytes to neuroinflammatory response.^29^ And this effect can only be maintained for a few days, we have also confirmed this situation after spinal cord injury in mice. Consistent with this previous study, we found that p52 was highly expressed in neurons. These results suggest that the noncanonical NF‐κB signaling pathway in astrocytes may participate in regulating neuroinflammation after SCI. ALX is a specific inhibitor of IKKε and TBK1 which increases phosphorylation of TBK1 on Ser172 and blocks polyinosinic: polycytidylic acid (poly I:C)‐stimulated phosphorylation of interferon responsive factor‐3 (IRF3).^13^ Previous studies confirm that TBK1 phosphorylates the transcription factor IRF3 to induce type‐I interferons and other cytokines.^30^ In addition, other studies have shown that the p‐IRF3 expression is upregulated in response to CNS injury and in astrocytes subjected to stretch injury,^11^ suggesting that the p‐IRF3 plays an important role in reactive astrogliosis.

Reactive astrogliosis is characterized by increased levels of glial fibrillary acidic protein (GFAP), release of inflammatory cytokines, and morphological changes in many neurological diseases.^31^ The reactive proinflammatory response of astrocytes, followed by the formation of glial scars and the inhibition of axonal regeneration, seems to be crucial.^32^ Previous studies have shown that reactive astrocytes can secrete multiple neurotoxic mediators^33^ and can also cause glial scarring, which isolates the lesion site preventing cellular repair and axonal regeneration.^34^ However, the role of glial scar in spinal cord injury and repair has been controversial. There is a view that suggests that astrocyte scar formation promotes axon regeneration after SCI.^35^ Taken all, reactive astrogliosis is considered a double‐edged sword. It may be responsible for the isolation of injured tissue from healthy tissue to prevent the spread of damage at the expense of blocking neuronal regeneration.^36^ Additionally, reactive astrogliosis has been demonstrated that it is involved in neurotoxicity, inflammation, and chronic pain. Recent studies indicated that suppressed neurotoxic astrogliosis by brain T_reg_ cells may promote the recovery of stroke and neuroinflammatory diseases.^37^ Our study shows that the levels of reactive astrogliosis and the inflammatory cytokines, IL‐1β, IL‐6, and TNF‐α, decreased in the SCI + ALX group after the SCI, which are linked to the neurological function scores. The inhibition of TBK1 activity following the ALX treatment inhibits the phenotypic shift of astrocytes from the resting state to the reactive state, as well as inhibiting astrocytic proliferation and reducing the expressions of inflammatory cytokines in vivo. The noncanonical NF‐κB signaling pathway has been extensively studied in the context of cancer initiation, antiviral immunity by regulating type I IFN expression.^38^ Recent studies show that injured spinal cords and astrocyte cultures can induce type I IFN production.^11^ In addition, levels of IRF3 protein are improved in astrocytes subjected to stretch injury and spinal cords subjected to moderate contusive injury.^11^ In our study, the noncanonical NF‐κB signaling pathway‐associated proteins, such as p100 and RelB, were significantly upregulated after SCI. The ALX treatment significantly reduced the expression of p‐IRF3 and its transcriptional activity in LPS‐stimulated astrocytes. These findings indicate that TBK1 offer novel therapeutic targets in neuroinflammatory and CNS injury by regulating innate immune response. The ALX treatment activated the noncanonical NF‐κB signaling pathway and inhibited p‐IRF3 activity following the SCI, thereby alleviating neuroinflammation.

However, how p‐IRF regulates reactive astrogliosis has rarely been studied. In our study, we found that the expression of p‐IRF3 protein was increased and peaked at 1 day after SCI, and p‐IRF3 upregulation was detected mainly in astrocytes and neurons. Meanwhile, recent studies have shown that the expression of IRF3 is positively correlated with that of Yes‐associated protein (YAP) in gastric cancer.^39^ YAP transcriptional coactivator is negatively regulated by the Hippo pathway, and it plays a key role in organ size control and tissue homeostasis.^40^ In addition, our previous research suggests that YAP promotes the formation of glia scars by positively regulating astrocytic proliferation after SCI. Similarly, we found that YAP activation was gradually induced after SCI 7 days.^17^ Meanwhile, we found that the ALX treatment inhibited the expression of p‐IRF3 and the nuclear translocation of YAP after SCI. Nuclear translocation plays a critical role in cellular biology as it enables the translocation of YAP into the nucleus, where it can bind to the transcriptional enhancer activator domain (TEA) domain transcription factor 4 to modulate the expression of genes that promote cell proliferation.^41^ Furthermore, IRF3 has been shown to interact with both YAP and TEAD4 within the nucleus, thereby enhancing their interaction and facilitating the nuclear translocation and activation of YAP.^39^ In this regard, our findings are relevant to TBK1/IRF3 signaling pathway involving in regulating reactive astrogliosis by alleviating YAP‐dependent astrocytic proliferation. Additionally, the massive release of inflammatory factors will further aggravate reactive astrogliosis in a pathological setting of injury to the spinal cord and stretch injury to astrocytes in culture. IRF3 and type I interferon (IFN) production hyperactivation promoted the pathological process of certain inflammatory diseases, such as MI‐associated inflammation.^42^ Previous researches have shown that interferon gamma (IFN‐γ) treatment promoted oligodendrocyte‐type‐2 astrocyte progenitor cell/oligodendrocyte precursor cell (O‐2A/OPC) differentiation of reactive astrocytes.^43^ Thus, activation of IRF3 could be a risk factor for SCI with the hyperactivation of reactive astrogliosis. Indeed, our results suggest that the inhibition of phosphorylation of IRF3 substantially inhibited reactive astrogliosis through downregulation of YAP activity. In particular, pharmacological inhibition of TBK1 by ALX inhibited the reactive astrogliosis with YAP hyperactivation.

TBK1 has been reported to be dispensable for the noncanonical NF‐κB signaling pathway activation on response to damage stimuli.^38^ The evidence on the crosstalk association of the YAP and the noncanonical NF‐κB pathways should be further investigated in future. Our research provides a novel relationship between the regulation of the SCI‐mediated reactive astrogliosis by the interactions of YAP and IRF3. The inhibitory effects of the ALX treatment on the inflammatory response and on the TBK1/YAP axis activation were similar to the depletion of IRF3 in astrocytes, suggesting that the anti‐inflammation effect of the ALX was associated with IRF3 expression.

The present study has several limitations. In our study, we focused on reactive astrocyte response to the ALX treatment after SCI. Due to the scope of our study and technical limitations, we cannot exclude the possibility that the TBK1 in neurons and other cells after injury plays a role in the repair of spinal cord injury. Actually, we have much interest in carrying out future studies relevant to this question. Further studies involving TBK1 knockout mice should be performed to investigate the mechanisms involved in the TBK1‐mediated regulation of YAP and the noncanonical NF‐κB pathway. Moreover, recent studies have identified that there is a sexual dimorphism in the propensity for the prognosis of CNS injury.^44^, ^45^ If, as is commonly thought, gene expression changes help dictate the pathological progress of the disease, and sexually dimorphic gene expression profiles may also influence it. For example, it was reported that 40% of the differential genes in rat brain micro‐vessels were concentrated in males, which were mainly related to nucleic acid binding, enzyme modulator, and transcription factor.^46^ In females, however, the differential genes were predominantly linked to mitochondrial function.^47^

In addition, sexual dimorphism in neurological function is also strongly associated with neurotransmitter delivery,^48^ cerebral blood flow,^49^ glial cell activation,^50^ and neuroinflammation.^51^ In the secondary injury cascade after spinal cord injury, among pathological microglia, female mouse microglia showed increased expression of ROS‐related genes, while male mouse microglia showed complement increased expression of C1qa genes of the pathway.^52^ This could lead to differences in the micro‐environment of the central nervous system between male and female individuals, and thus different responses to disease. Although the current study only used male animals, in future studies, we should expand to both sexes to determine whether the effect of TBK1 inhibition is independent of sex.

## 5 CONCLUSIONS

Our results show that TBK1‐medicated innate immune responses play an important role in the activation of reactive astrocytes after SCI. Inhibition of TBK1 can promote the activation of noncanonical NF‐κB signaling pathways and inhibit phosphorylation of IRF3. More importantly, we found that the expression of phosphorylation of IRF3 participates in regulating YAP nuclear translocation in LPS‐induced astrocyte inflammation model. Collectively, our study supports the view that inhibition of TBK1 can alleviate neuroinflammation after SCI and ALX may be a potential therapeutic drug for SCI.

## AUTHORS' CONTRIBUTION

**Wenbin Zhang**: Conceptualization, investigation, data curation, and writing‐original draft. **Mengxian Jia** and **Jiashu Lian**: Investigation, data curations, and writing‐original draft. S**heng Lu**, **Jian Zhou** and **Ziwei Fan**: Methodology, validation, and data analysis. **Zhoule Zhu**, **Yaozhi He** and **Changgang Huang**: Investigation and formal analysis. **Mingyu Zhu** and **Jian Wang**: Methodology and resources. **Ying Wang** and **Zhihui Huang**: Supervision, funding acquisition, writing‐review and editing. **Honglin Teng**: Conceptualization, supervision, funding acquisition, and writing‐review and editing.

## CONFLICT OF INTEREST STATEMENT

The authors declare that they have no competing interests.

## Supporting information


Figure S1

Figure S2

Figure S3

Figure S4

## Data Availability

The data used to support the findings of this study are available from the corresponding author upon request.
