# Supplementary figures and images for: Inhibition of TANK‐binding kinase1 attenuates the astrocyte‐mediated neuroinflammatory response through YAP signaling after spinal cord injury
Source: CNS Neurosci Ther. 2023 Apr 10;29(8):2206–22. doi: 10.1111/cns.14170 (PMC10352897; doi:10.1111/cns.14170)

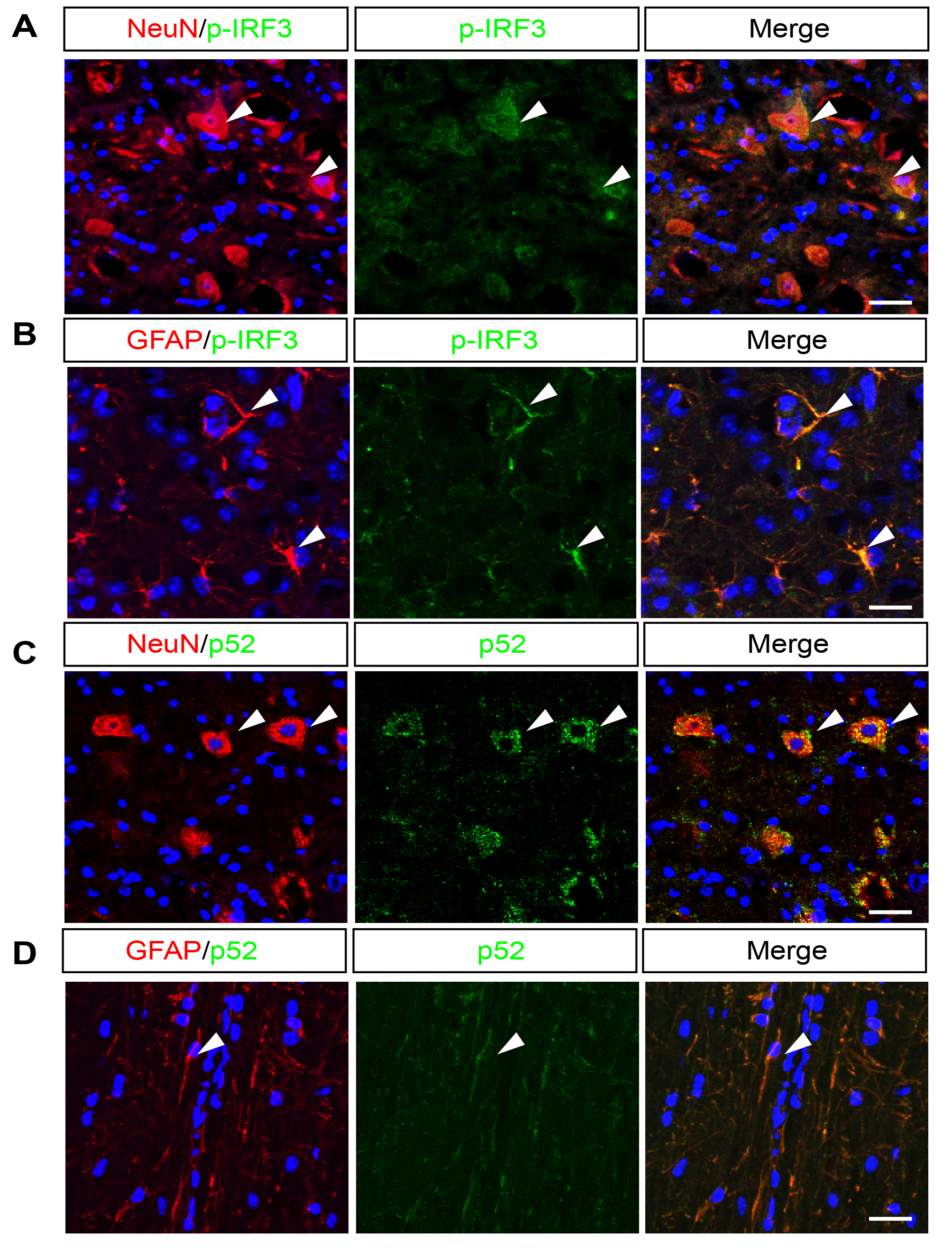

Supplement: Supplementary file 1 — Figure S1 [file CNS-29-2206-s002.jpg]

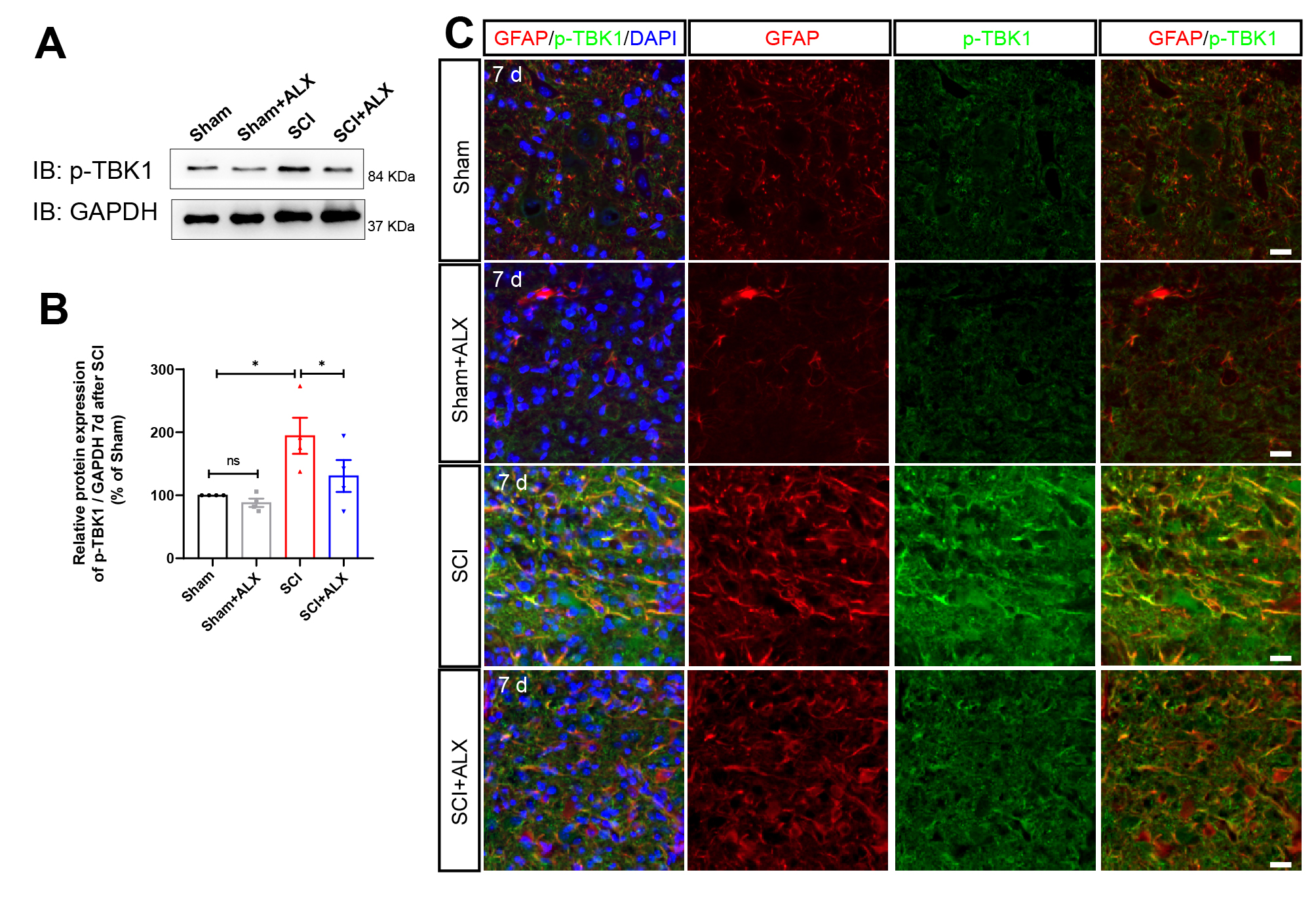

Supplement: Supplementary file 2 — Figure S2 [file CNS-29-2206-s003.jpg]

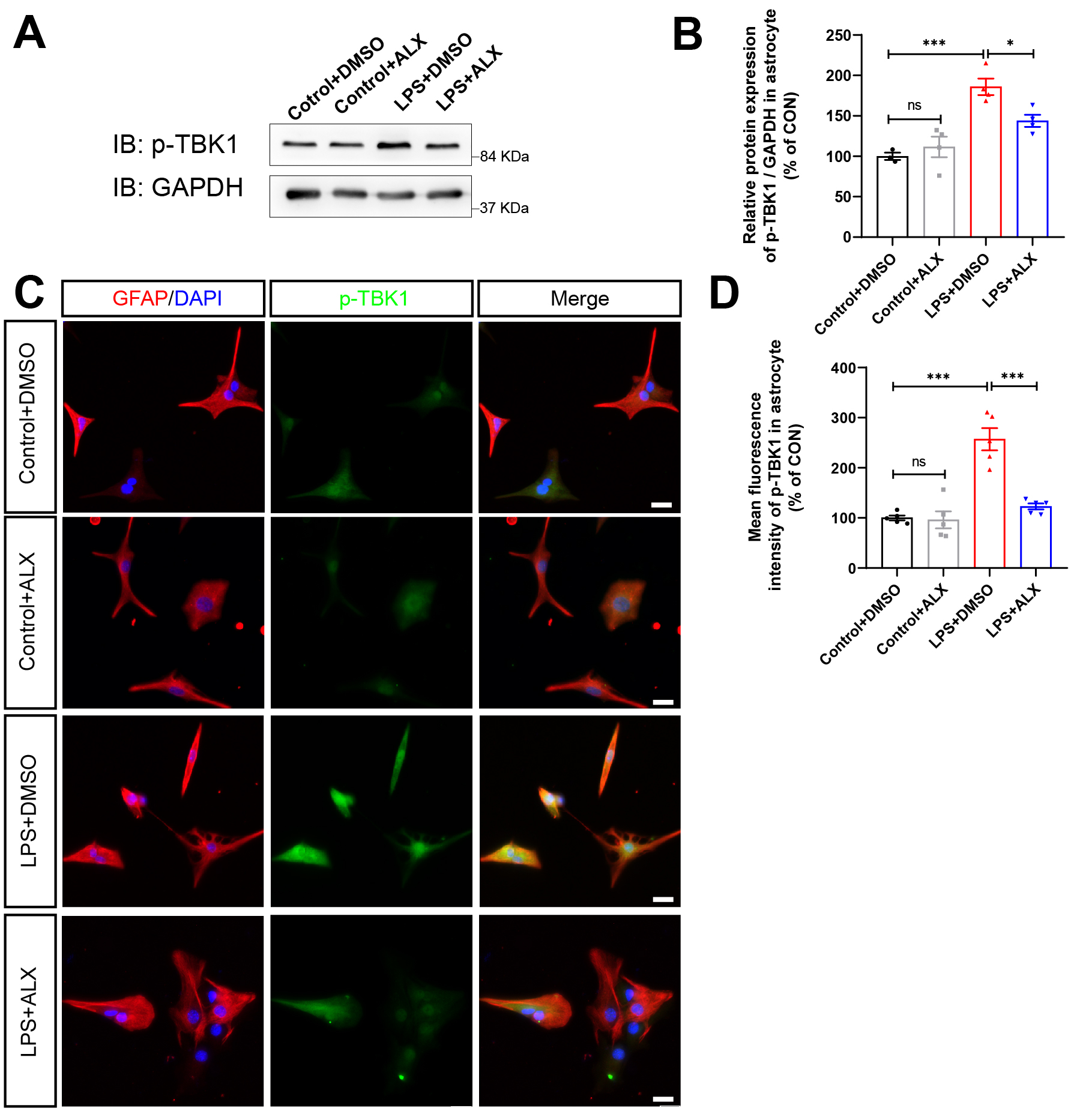

Supplement: Supplementary file 3 — Figure S3 [file CNS-29-2206-s001.jpg]

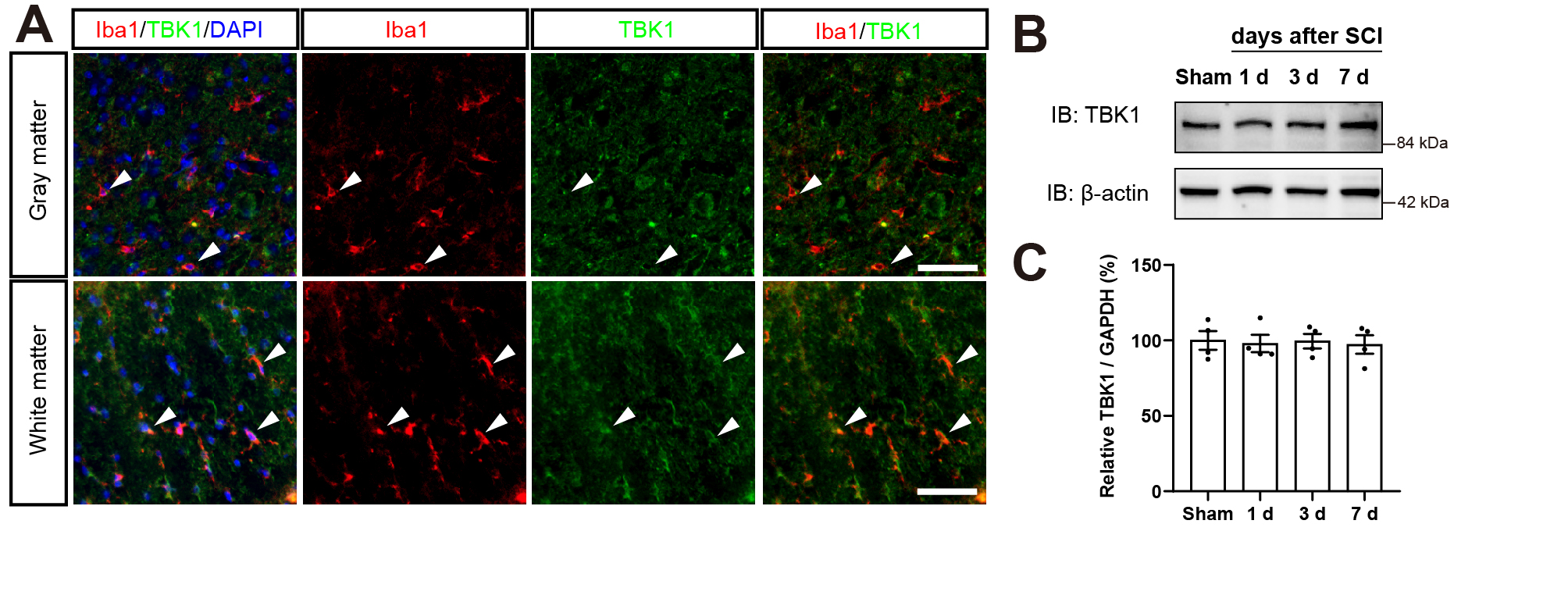

Supplement: Supplementary file 4 — Figure S4 [file CNS-29-2206-s004.jpg]
